# GI bleeding in patients with left ventricular assist device: endoscopic approach and prediction model using supervised machine learning

**DOI:** 10.1016/j.igie.2023.07.016

**Published:** 2023-07-30

**Authors:** Dan McEntire, Benjamin Gow-Lee, Kimberly Kucharski, Hassam Ali, John Fang, Babu P. Mohan

**Affiliations:** 1 Advanced Gastroenterology, Virginia Commonwealth University, Virginia, USA; 2 Internal Medicine, University of Utah, Salt Lake City, USA; 3 Gastroenterology & Hepatology, East Carolina University/Brody School of Medicine, Greenville, North Carolina, USA; 4 Gastroenterology & Hepatology, University of Utah, Salt Lake City, Utah, USA; 5 Mohan: Gastroenterology, Orlando Gastroenterology PA, Orlando, Florida, USA

## Abstract

**Background and aims:**

GI bleeding is a common adverse event in patients with left ventricular assist devices (LVADs). Data are limited regarding the optimal endoscopic management approach and quantifiable risk of bleeding.

**Methods:**

This retrospective analysis included adult patients who underwent LVAD implantation at the University of Utah between February 1996 and September 2021. The endoscopic management of GI bleeding events was analyzed, along with clinical predictors of bleeding, using a supervised machine learning model.

**Results:**

A total of 557 LVADs were implanted during the study period. Of these, 132 (23.7%) had at least 1 GI bleed, with a total of 252 GI bleeds. Gastric (17.2%) and non-duodenum small intestinal (13.1%) bleeding were the most common sites. A total of 429 procedures were performed; EGD was the most common (45.5%). Video capsule endoscopy exhibited the highest diagnostic yield overall (68.9%); 203 (80.6%) GI bleeds were treated with endoscopy (EGD, 45.5%; colonoscopy, 26.8%). A hemostatic intervention was possible in 32.8% of EGDs, 22.6% of colonoscopies, and 26.2% of push enteroscopies. For the model predicting GI bleeding risk after LVAD transplantation, 3 predictors were retained: destination therapy (score = 1.5), warfarin use (score = 5), and antiplatelet use (score = 10). A total score ≥10 was considered high risk.

**Conclusions:**

Although EGD was the most performed procedure, push enteroscopy rivaled both EGD and colonoscopy on diagnostic yield and percentage of interventions performed. Clinical variables of destination therapy, warfarin use, and antiplatelet use were retained by the machine learning model to help quantify risk of GI bleed.

Mechanical left ventricular assist devices (LVADs) are an established treatment for advanced heart failure.^1^ LVADs require anticoagulation and antiplatelet therapy to avoid in-device thrombosis and potential catastrophic embolism. GI bleeds are a major adverse event of LVAD therapy, with >20% of patients experiencing a GI bleed within the first 12 months and >40% after 60 months.^1^ Many of these GI bleeds can be life-threatening due to anticoagulation use and a precarious hemodynamic status in this patient population.

Based on published literature thus far, the most common reported cause of GI bleed in patients with LVAD are angiodysplasias or arteriovenous malformations.^2^, ^3^, ^4^ A bleeding source, if found, is most often reported in the stomach (30%), followed by the small bowel (21%), then colon (19%).^2^^,^^4^ A source is not found in up to one-quarter of GI bleeds.^2^ Current data are limited by small sample size, and major GI society guidelines do not make specific recommendations on the endoscopic management of GI bleed in patients with LVAD.^2^^,^^3^

The goal of the current study was to describe the characteristics of LVAD patients with GI bleeding cared for at an academic medical center and associated Veterans Affairs medical center with a large LVAD program. In particular, the intention was to describe the endoscopic management of GI bleeding in patients with LVAD and propose an optimal endoscopic management approach in this patient population based on our large-center experience. In addition, we aimed to propose a prediction model using supervised machine learning on the risks of GI bleed in patients with LVAD.

## Methods

### Study population

This retrospective cohort study included prospectively collected and maintained data of all adult patients (age >18 years) who had an LVAD implanted at our institution (University of Utah Health, Salt Lake City, Utah, USA) between February 1996 and September 2021. GI bleeding events that were evaluated and treated at the University of Utah Hospital and/or George E. Wahlen Veteran Affairs Medical Center were included in the analysis. GI bleeding was defined based on overt clinical presentation consistent with possible bleeding in the GI tract such as melena, hematochezia, and hematemesis in patients with LVAD. Patients with possible occult bleeding were not included. The study protocol was approved by the University of Utah institutional review board and was exempt due to retrospective de-identified data.

### Study data

Data were collected for patients with LVAD placement being prospectively admitted for GI bleeding. Each GI bleed event was recorded, and data were collected until death, heart transplantation, or end of follow-up time. Data collected included patient demographic characteristics, LVAD type, indication for LVAD, duration of LVAD therapy, presence or absence of GI bleed, total number of GI bleeds, admission length, anticoagulation or antiplatelet medication use on presentation, baseline hemoglobin and international normalized ratio values, and number of units of packed red blood cells transfused. Endoscopic data collected included the type of procedure(s) performed and whether it was performed as an initial procedure or follow-up to an earlier procedure, date performed, bleeding sources identified, and any hemostatic interventions. Cases were excluded if patient age was <18 years or if data of interest were incomplete.

### Study outcomes

The primary outcome of interest was the diagnostic yield of various endoscopic procedures, defined as the proportion of successful identification of a bleeding source, and proportion of endoscopic interventions performed in the management of GI bleed in patients with LVAD. Data were gathered and segregated based on EGD, push enteroscopy, colonoscopy, video capsule endoscopy (VCE), flexible sigmoidoscopy (FS), and single-balloon enteroscopy (SBE). GI bleed cases with index (initial) procedure and secondary approach procedures (when the primary index procedure failed to address the GI bleed) were noted. The proportion of diagnostic yield on endoscopy and interventions performed was calculated for the index procedure and secondary procedure. The secondary goal of this analysis was to use a supervised machine learning approach on patients’ clinical variables to propose a prediction model to identify and quantify high risk for GI bleed in patients with LVAD.

### Statistical analysis

Descriptive rates of GI bleeding outcomes of interest and endoscopic data were calculated as percentage values. Statistical analysis was performed by using STATA version 16 (StataCorp LLC, College Station, Tex, USA). The derivation of a risk scoring system by supervised machine learning and nomogram development is detailed in the Appendix 1 (available online at www.igiejournal.org).

## Results

### Patient characteristics

A total of 574 patients were identified, with 17 excluded due to incomplete data. Of the 557 patients included in this study, 132 (23.7%) patients had at least 1 GI bleed, and 64 (11.5%) had multiple bleeds. In total, 252 GI bleed events were treated at our facilities. Each occurrence was considered as a separate GI bleeding event for the purpose of this study. A total of 486 (84%) patients were male. The median age of the study cohort was 59 years, and the median hospital length of stay (LOS) per event was 9 days. The average number of days from device implantation to overt GI bleeding was 338 days. Baseline patient characteristics are summarized in Table 1.

**Table 1:** Baseline patient characteristics

Factor	No GI bleed	GI bleed	*P* value
No. of patients	425	132	
Age, median (IQR), y	59 (49-66)	59.5 (50-66)	.64
Age groups			.20
18-34 y	37 (10.3%)	11 (8.6%)	
34-49 y	48 (13.4%)	20 (15.6%)	
50-64 y	167 (46.5%)	55 (43.0%)	
65-79 y	96 (26.7%)	42 (32.8%)	
≥80 y	11 (3.1%)	0	
Sex			.50
Female	48 (13.2%)	20 (15.6%)	
Male	315 (86.8%)	108 (84.4%)	
Duration of LVAD implantation, mo			.37
0-12	165 (28.4%)	62 (48.44%)	
13-24	47 (17.1%)	21 (16.41%)	
>24	151 (41.6%)	45 (35.2%)	
LOS for GI bleed admission, median (IQR), d	0 (0-0)	8.0 (3.0-17.0)	<.001
Continuous or pulsatile flow LVAD			.062
Continuous	224 (95.7%)	79 (100%)	
Pulsatile	10 (4.3%)	0	
Taking any anticoagulant on admission	3 (0.8%)	108 (83.37%)	<.001
Type of anticoagulant on admission			
Warfarin	3 (0.8%)	82 (64.1%)	<.001
Heparin	0	22 (17.2%)	<.001
Enoxaparin	0	4 (3.1%)	<.001
Indication for LVAD transplantation			.069
Bridge to decision	89 (24.5%)	29 (22.7%)	
Bridge to recovery	1 (.3%)	0	
Bridge to transplantation	151 (41.6%)	40 (31.3%)	
Destination therapy	122 (33.6%)	59 (46.1%)	
Any antiplatelet use on admission for GI bleed	3 (.8%)	95 (74.2%)	<.001
DAPT on admission for GI bleed	0	7 (5.5%)	<.001
Clopidogrel/ticagrelor/prasugrel use on admission for GI bleed	0	4 (3.1%)	<.001
Dipyridamole use on admission for GI bleed	0	4 (3.1%)	<.001
Median hemoglobin on admission (IQR), g/dL	6.3 (5-8.3)	7.6 (6.7-8.8)	.21
Median INR on admission (IQR)	3.8 (3-3.9)	2.4 (1.6-4.1)	.08
Median GI bleed episodes (IQR)	0 (0-0)	1 (1-3)	<.001

*IQR*, Interquartile range; *LVAD*, left ventricular assist device; *LOS*, length of stay; *DAPT*, dual antiplatelets; *INR,* international normalized ratio.

### Endoscopic approach

A total of 203 (80.6%) GI bleed events were treated with at least 1 form of endoscopy, and the cumulative number of endoscopy procedures performed was 429. The choice of initial endoscopy procedure was based on bedside clinical decision-making per the patient’s clinical presentation. Stable patients with melena underwent EGD first, as did unstable patients presenting with hematochezia. Hemodynamically stable patients with hematochezia were offered a lower-endoscopy procedure first. Multiple endoscopy procedures were needed due to inability to locate or identify the culprit bleeding lesion on index procedure. The total number of EGDs performed was 195 (45.5%) of all 429 procedures; colonoscopy, 115 (26.8%); push enteroscopy, 65 (15.2%); VCE, 45 (10.4%); SBE, 5 (1.2%), and FS 4 (0.9%) (Table 2).

**Table 2:** Summary of endoscopic approach

	EGD		Colonoscopy		Push enteroscopy		VCE		SBE		FS	
	n	%	n	%	n	%	n	%	n	%	n	%
Total procedures (N = 429)	195	45.5	115	26.8	65	15.2	45	10.4	5	1.2	4	0.9
GI bleeds (n = 252) undergoing at least 1 procedure	137	54.4	95	37.7	58	23.0	45	18.0	5	2.0	4	1.6
GI bleeds (n = 252) undergoing >1 procedure	58	23.0	20	7.9	7	2.8	0	0	0	0	0	0
GI bleeds (n = 252) having procedure selected as initial choice	129	51.2	32	12.7	39	15.5	5	2.0	1	0.4	3	1.2
GI bleeds (n = 252) having procedure performed as a subsequent/secondary study	65	25.8	83	32.9	26	10.3	40	15.9	4	1.6	1	0.4

*VCE*, Video capsule endoscopy; *SBE*, small-bowel enteroscopy; *FS*, flexible sigmoidoscopy.

Among all GI bleeding events (N = 252), at least 1 EGD was performed in 137 (54.4%), and 58 (23%) had multiple EGDs. Ninety-five (37.7%) had at least 1 colonoscopy performed, and 20 (7.9%) had multiple colonoscopies. Fifty-eight (23%) had at least 1 push enteroscopy performed, and 7 (2.8%) had multiple push enteroscopies; 45 (18%) had 1 VCE, 5 (2%) had 1 SBE, and 4 (1.6%) had 1 FS.

Among all GI bleeding events (N = 252), EGD was selected as the initial endoscopic study in 129 (51.2%), colonoscopy in 32 (12.7%), push enteroscopy in 39 (15.5%), VCE in 5 (2.0%), SBE in 1 (0.4%), and FS in 3 (1.2%). SBE and FS were chosen as initial modalities in patients presenting with hematochezia. Eight-five (33.7%) GI bleed events were treated with >1 endoscopic procedure. Among all GI bleeding events (252) in which multiple procedures were performed, EGD was performed as a secondary/subsequent procedure in 65 (25.8%), colonoscopy in 83 (32.9%), push enteroscopy in 26 (10.3%), VCE in 40 (15.9%), SBE in 4 (1.6%), and FS in 1 (0.4%).

### Diagnostic yield of endoscopy

Collectively, 215 (50.1%) of 429 total endoscopic procedures identified at least 1 GI bleeding site (Table 3). A total of 108 (55.4%) of all 195 EGDs identified at least one bleeding site. When performed as the initial endoscopic procedure, 65 (33.3%) identified a bleeding source. When performed as a secondary study, 43 (22.1%) identified a bleeding site, and 47 (40.9%) of all 115 colonoscopies identified at least 1 bleeding site. When performed as the initial endoscopic procedure, 20 (17.4%) identified a bleeding source. When performed as a secondary study, 27 (23.5%) identified a bleeding site.

**Table 3:** Summary of diagnostic yield of endoscopy

	EGD (n = 195)		Colonoscopy (n = 115)		Push enteroscopy (n = 65)		VCE (n = 45)		SBE (n = 5)		FS (n = 4)	
	n	%	n	%	n	%	n	%	n	%	n	%
Any bleeding site identified	108	55.4	47	40.9	25	38.5	31	68.9	1	20.0	3	75.0
Any bleeding site identified, when performed as the initial procedure	65	33.3	20	17.4	22	33.8	3	6.7	0	0	2	50.0
Any bleeding site identified, when performed as a subsequent/secondary study	43	22.1	27	23.5	3	4.6	28	62.2	1	20.0	1	25.0

*VCE*, Video capsule endoscopy; *SBE*, small-bowel enteroscopy; *FS*, flexible sigmoidoscopy.

Twenty-five (38.5%) of all 65 push enteroscopies identified at least 1 bleeding site. When performed as the initial endoscopic procedure, 22 (33.8%) identified a bleeding source. When performed as a secondary study, 3 (4.6%) identified a bleeding site. Thirty-one (68.9%) of all 45 VCEs identified at least 1 bleeding site. When performed as the initial endoscopic procedure, 3 (6.7%) identified a bleeding source. When performed as a secondary study, 28 (62.2%) identified a bleeding site.

One (20%) of the 5 SBE procedures performed identified at least 1 bleeding site and had been performed as a secondary study. Three (75%) of all 4 FS identified at least 1 bleeding site. When performed as the initial endoscopic procedure, 2 (50%) identified a bleeding source. When performed as a secondary study, 1 (25%) identified a bleeding site.

### Bleeding sites

Collectively, the site of GI bleeding was localized to the esophagus in 28 (6.5%) of 429 total procedures, stomach in 74 (17.2%), duodenum in 23 (5.4%), non-duodenum small intestine in 56 (13.1%), and colon in 51 (12.1%) (Table 4).

**Table 4:** Summary of bleeding sites

	EGD (n = 195)		Colonoscopy (n = 115)		Push enteroscopy (n = 65)		VCE (n = 45)		SBE (n = 5)		FS (n = 4)		Total (N = 429)	
Esophagus	28	14.4%	NA		0	0%	0	0%	0	0%	NA		28	6.5%
Stomach	63	32.3%			9	13.8%	2	4.4%	0	0%			74	17.2%
Duodenum	11	5.6%			8	12.3%	4	8.9%	0	0%			23	5.4%
Small intestine (non-duodenum)	NA		14	12.2%	13	20.0%	28	62.2%	1	20.0%			56	13.1%
Colon			47	40.9%	NA		1	2.2%	NA		3	75.0%	51	12.1%

*VCE*, Video capsule endoscopy; *SBE*, small-bowel enteroscopy; *FS*, flexible sigmoidoscopy; *NA*, not applicable.

A total of 195 total EGDs localized a bleeding site to the esophagus in 28 (14.4%), stomach in 63 (32.3%), and duodenum in 11 (5.6%); 115 total colonoscopies localized a bleeding site to the colon in 47 (40.9%). In addition, when blood or hematin was identified in the terminal ileum without a colonic bleeding source, small-bowel bleeding was suspected. Thus, small-bowel blood loss was identified in 14 (12.2%) colonoscopies.

Sixty-five total push enteroscopies localized a bleeding site to the esophagus in 0 (0%), stomach in 9 (13.8%), duodenum in 8 (12.3%), and non-duodenum small intestine in 13 (20%). Forty-five total VCE procedures localized a bleeding site to the esophagus in 0 (0%), stomach in 2 (4.4%), duodenum in 4 (8.9%), non-duodenum small intestine in 28 (62.2%), and colon in 1 (2.2%). Five total antegrade SBE procedures localized a bleeding site to the esophagus in 0 (0%), stomach in 0 (0%), duodenum in 0 (0%), and non-duodenum small intestine in 1 (20%). Four total FS procedures localized a bleeding site to the colon in 3 (75%).

### Endoscopic interventions

Collectively, hemostatic interventions were performed in 108 (25.2%) of 429 total procedures. When the procedure had been performed as the initial study, 58 (13.5%) were accompanied by an intervention. When the procedure had been performed as a secondary/subsequent study, 50 (11.7%) were accompanied by an intervention (Table 5).

**Table 5:** Summary of endoscopic intervention

	EGD (n = 195)		Colonoscopy (n = 115)		Push enteroscopy (n = 65)		VCE (n = 45)	SBE (n = 5)		FS (n = 4)		Total (N = 429)	
	n	%	n	%	n	%		n	%	n	%	n	%
Intervention performed	64	32.8	26	22.6	17	26.2	NA	1	20.0	0	0	108	25.2
Intervention when performed as initial procedure	34	17.4	13	11.3	11	16.9		0	0	0	0	58	13.5
Intervention when performed as a subsequent/secondary study	30	15.4	13	11.3	6	9.2		1	20.0	0	0	50	11.7

*VCE*, Video capsule endoscopy; *SBE*, small-bowel enteroscopy; *FS*, flexible sigmoidoscopy; *NA*, not applicable.

Of a total of 195 EGDs performed, 64 (32.8%) resulted in hemostatic intervention. When EGD had been performed as the initial study, 34 (17.4%) were accompanied by an intervention. When the EGD had been performed as a secondary/subsequent study, 30 (15.4%) were accompanied by an intervention.

Twenty-six (22.6%) colonoscopies of a total of 115 performed resulted in hemostatic intervention. When the colonoscopy had been performed as the initial study, 13 (11.3%) were accompanied by an intervention. When the colonoscopy had been performed as a secondary/subsequent study, 13 (11.3%) were accompanied by an intervention.

Seventeen (26.2%) push enteroscopies of a total of 65 performed resulted in hemostatic intervention. When push enteroscopy had been performed as the initial study, 11 (16.9%) were accompanied by an intervention. When the push enteroscopy had been performed as a secondary/subsequent study, 6 (9.2%) were accompanied by an intervention.

By definition, no intervention was performed by VCE. One (20%) SBE of a total of 5 performed resulted in hemostatic intervention, and this procedure had been completed as a secondary/subsequent procedure. None of the total 4 FSs performed resulted in any hemostatic intervention.

### GI bleed versus no GI bleed

There was no difference in LVAD device types/ brands in either cohort (*P* = .42) (Table 1). Warfarin was used more in patients with a GI bleed (64.1%). There was a higher frequency of device-related deaths in patients with GI bleed compared with no GI bleed (45.3% vs 30.4%, *P* = .002). Device-related deaths were secondary to cardiac arrhythmias, heart failure, and device thrombosis.

In patients with GI bleeding, the median LOS for hospitalization was 25 days (interquartile range, 14-39 days), and in patients without GI bleeding, it was 19 days (interquartile range, 10-32 days) (*P* = .002). There was a higher prevalence of continuous-flow LVAD in the GI bleed cohort compared with the no GI bleed cohort (100% vs 95.7%, *P* = .062). There was a higher prevalence of anticoagulant use on admission (83.37% vs 0.8%) and antiplatelet use on admission (74.2% vs 0.8%) in the GI bleed cohort compared with the no GI bleed cohort (*P* < .001). There was a higher prevalence of LVAD indication as destination therapy in the GI bleed cohort (46.1% vs 33.6%) compared with the cohort without GI bleeding (100% vs 95.7%, *P* = .069).

### Predictive model for GI bleed

Initial screening using univariate regression shortlisted 11 variables with a positive association to GI bleeding: anticoagulant use, antiplatelet use, warfarin use, dual antiplatelet use, dipyridamole use, clopidogrel use, heparin use, enoxaparin use, age groups, sex, and implant indications. Multicollinearity was assessed, and the first model revealed a variance inflation factor >10 for “on any anticoagulant” and “on any antiplatelet” indicating strong evidence of multicollinearity. The exclusion of the variable “any antiplatelet use” did not eliminate it. However, excluding “any anticoagulant use” resulted in a suitable balance of predictors without evidence of multicollinearity (Supplementary Table 1, available online at www.igiejournal.org).

**Figure 1:**
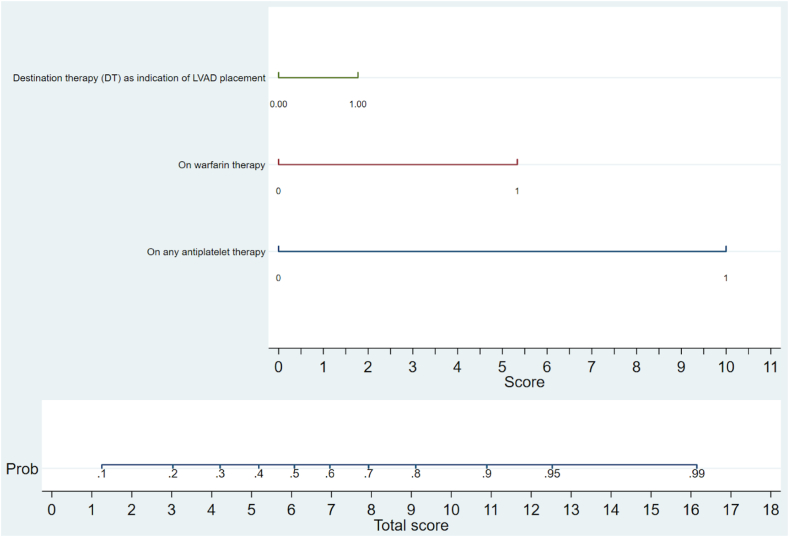
Risk prediction nomogram for GI bleeding in left ventricular assist device (LVAD) patients incorporating warfarin use (1 = yes; 0 = no), acute antiplatelet use (1 = yes; 0 = no), and destination therapy as indication of LVAD placement (1 = yes; 0 = no).

The selected variables underwent least absolute shrinkage and selection operator (LASSO) regression, resulting in the retention of 6 predictors for GI bleeding risk in LVAD patients, with a mean lambda of 0.02. They included age <34 years, female sex, warfarin use, bridge to transplantation/destination therapy as transplant indication, and any antiplatelet use. A multivariable logistic model was created for nomogram development after further omitting variables with negative regression coefficients indicating poor association with outcome (Supplementary Tables 2 and 3, available online at www.igiejournal.org). The remaining 3 predictors (destination therapy as an indication of LVAD placement and any antiplatelet or warfarin use) were used to develop a nomogram for predicting GI bleeding risk in LVAD patients (Supplementary Table 4, available online at www.igiejournal.org). The nomogram had 1 scale corresponding to each predictor and their score, 1 total score, and 1 probability scale. The score assigned was 1.5 for destination therapy as an indication of LVAD placement, 5 for warfarin use, and 10 for any antiplatelet use. The nomogram is illustrated in Figure 1.

**Supplementary Figure 1:**
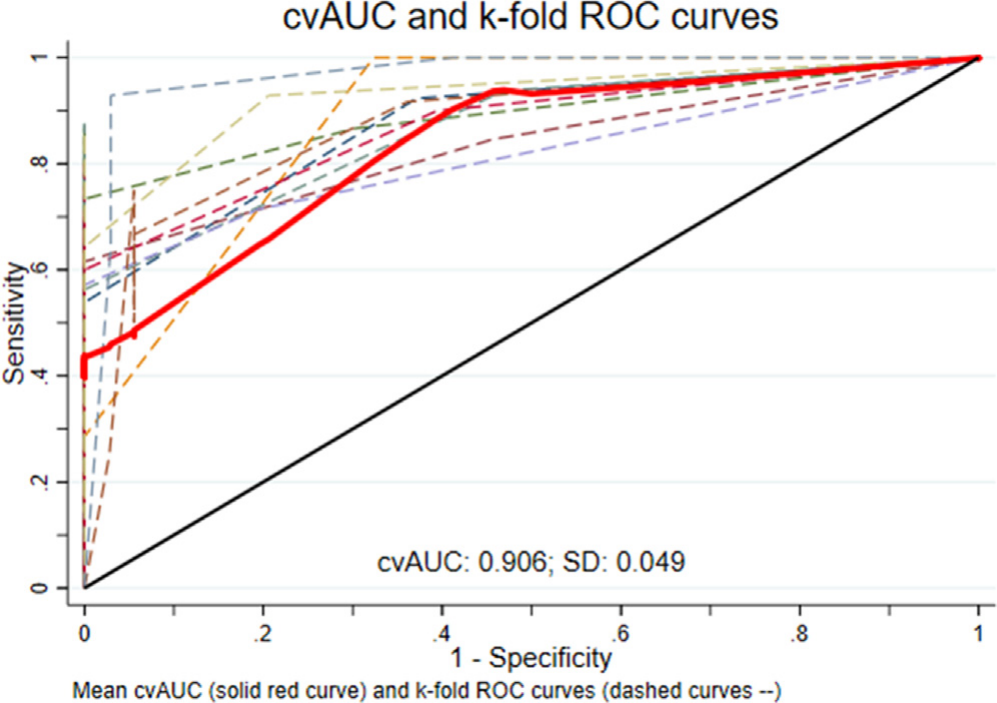
Receiver-operating curve (ROC) with mean cross-validated area under the curve (CvAUC) after 10-fold-cross validation. AUC = .9057 (bias-corrected bootstrap 95% confidence interval, .8477-.9323). *SD*, Standard deviation.

**Supplementary Figure 2:**
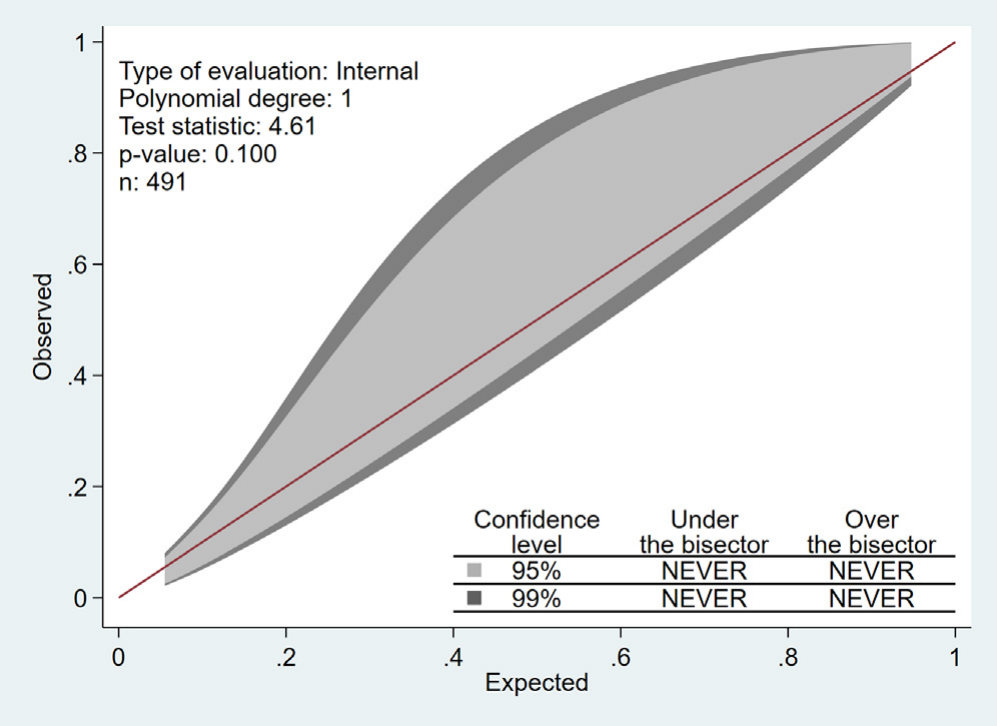
Calibration belt reporting no area of miscalibration at the 95% over the bisector or under the bisector and no miscalibration over or under the bisector at 99% confidence levels.

The nomogram underwent 10-fold cross-validation to generate the final model’s mean area under the curve (AUC) (cross-validated mean) and internal validation of the risk score. The AUC of the final model was .9057 (bootstrap bias-corrected 95% confidence interval, .8477-.9323), which correlated to a 90% probability of the model correctly assigning a higher risk score to patients at risk of GI bleeding after LVAD placement (Supplementary Fig. 1, available online at www.igiejournal.org). The calibration belt plot reported no significant miscalibration at the 95% and 99% confidence levels (Supplementary Fig. 2, available online at www.igiejournal.org). The *P* value was insignificant (test statistic = 4.61; *P* = .10), demonstrating no miscalibration. The Brier score was .06.

The model predicted risk scores ranged from 4.56% to 94.74%. Liu’s index was used to determine the cutoff at 10 points. Patients with a score ≥10 were therefore considered high risk with a sensitivity of 74.22% and a negative predictive value of 96.94%; the specificity was 99.15% with a positive predictive value of 91.36%.

## Discussion

In this large retrospective study, the overall rate of at least 1 GI bleed event was 23.7%. The culprit lesion was in the stomach in 17.2% of cases, followed by non-duodenum small intestinal bleeding in 13.1%. Of these, 80.6% underwent an endoscopy, with EGD being the most performed first-line procedure. To the best of our knowledge, the current study summarizing GI bleed and endoscopic management data from 557 patients with LVAD is the largest in the literature thus far.

Although our data are similar to those of published studies in terms of the etiology of GI bleed, key observations were made with respect to the endoscopic management of GI bleed in patients with LVAD.^4^ Procedural numbers for FS and antegrade SBE are very low in this study and are largely excluded from the discussion. Thus, the overall diagnostic yield was greatest in VCE (68.9%), although the large majority of these were performed as a secondary study, the patients having already had other endoscopic procedures. Notably, when a procedure was selected as the first-line study for a given bleeding event, push enteroscopy (33.8%) was most likely to identify a culprit lesion, followed closely by EGD (33.5%). When multiple procedures were needed to identify a source, VCE (62.2%) was again the most likely procedure to yield a site, followed by colonoscopy (23.5%). This suggests a potentially high diagnostic value for VCE in the LVAD GI bleeding population.

The most common bleeding sites were the stomach (17.2%) and the non-duodenal small intestine (13.1%). Overall, just 25.2% of all procedures resulted in hemostatic intervention. Of these, 32.8% of all EGDs resulted in hemostatic intervention, followed by push enteroscopy in 26.2%. The similarity between these 2 procedures, in the context of the most common bleeding sites, is of particular relevance given that diagnoses and interventions performed at EGD can also be performed at push enteroscopy. Detailed extraction of data on endoscopic interventions was not performed as it was out of scope for the current study.

Chances of ascertaining a cause of GI bleeding were higher with VCE compared with other endoscopic procedures, which was especially true when other endoscopic procedures failed to yield a GI lesion in the inpatient setting. Depending on clinical circumstances, VCE may be the most appropriate test to identify GI bleeding in this cohort, especially if the intention is medical treatment (eg, anticoagulation adjustment, use of adjunctive therapies) rather than endoscopic management, especially where sedation risks are believed to be high. In a majority of inpatient admissions, however, definitive management with endoscopic intervention may be the preferred outcome. In this circumstance, our data suggest that proceeding directly with push enteroscopy as the initial procedure in this cohort is warranted. This approach may negate the need for any or multiple EGD procedures, especially in circumstances in which results of the first EGD were negative. In the scenario in which a push enteroscopy is selected as the first procedure and fails to yield a diagnosis or result in hemostatic intervention, our data suggest colonoscopy as the next procedure of choice.

In the prediction model analysis using supervised machine learning, outstanding performance of the risk prediction nomogram was shown with no miscalibration. The supervised machine learning algorithm retained 3 predictors, namely, destination therapy as an indication of LVAD placement (score = 1.5), warfarin use (score = 5), and any antiplatelet use (score = 10). The AUC of the final model correlated to a 90% probability of the model correctly assigning a higher risk score to patients at risk of GI bleeding after LVAD placement. We suggest that the nomogram may be useful clinically for predicting patients at high risk for GI bleed in those with an LVAD. Higher-risk patients could be monitored and managed more closely, perhaps decreasing the need for prolonged inpatient admission. As shown by our data, LVAD patients with GI bleeding had longer median LOS versus patients without GI bleed and a greater frequency of device-related deaths.

Patients with a score ≥10 were considered high risk with a sensitivity of 74% and a negative predictive value of 97%; the specificity was 99% with a positive predictive value of 91%. The proposed nomogram can be used in 3 steps. First, the value corresponding to a patient for each predictor is read, and the score scale is used to calculate the scores. The total score is then obtained by adding individual scores calculated in the previous step. Finally, the bottom scale with the total score and probabilities scale is used to assess the probability of an event happening; for example, if a patient underwent LVAD placement as a destination therapy and was discharged only on warfarin (no antiplatelets), then the application of the nomogram yields a score of 6.5 (5 for warfarin and 1.5 for destination therapy), which corresponds to a 55% risk of GI bleeding. The patient is not taking any antiplatelets. The application of the nomogram yields a score of 6.5 (5 for warfarin and 1.5 for destination therapy), which correlates to a 55% risk of GI bleeding. We hope the proposed nomogram can be used to counsel patients with a quantifiable prediction value and help strategize the clinical and endoscopic management leading to timely intervention. However, the prediction nomogram currently does not help with making clinical adjustments in a proactive manner. We hope, with additional data on GI lesions and therapeutic interventions, that the scope of the nomogram could be expanded in ascertaining the type of lesion and ideal endoscopy intervention for better clinical outcome.

There are limitations in the current study, many inherent to the nature of retrospective studies. Data were not available with respect to stopping or continuing the antiplatelets and/or anticoagulants during inpatient admission; however, based on our single-center experience, many of these patients had these medications held or adjusted upon admission for GI bleed. We have used available predictors to derive the risk model, although some predictors might have been missed. The AUC of 90% has outstanding predictability and internal validity; however, there is a need for external validity to optimize its precision in making clinical judgments. In addition, patients who had incomplete data were excluded, which may have limited generalizability for those cases. Certain variables could be significantly associated with GI bleeding risk but, because of lack of predictive power, were automatically excluded after LASSO regression. Finally, the use of current data in a retrospective analysis is subject to bias inherent to retrospective studies, although all data were collected prospectively.

Nevertheless, despite these limitations, strengths of this study reside in the reported endoscopic management data for GI bleed in patients with LVAD. We used a vigorous 10-fold cross-validation for internal validation, which delivers a lower bias of estimates than split-sample validation, resulting in more sure predictions.^5^ There was no miscalibration assessed by the calibration belt, hence no likelihood of overfitting and good generalizability of the model. To the best of our knowledge, this study is the first to report on the endoscopic approach and derive a machine learning–based prediction model for GI bleed in patients with LVAD. Finally, as in any clinical situation, the initial endoscopic procedure must be tailored to the patient's clinical presentation of upper or lower GI bleed with attention to hemodynamic status.

In conclusion, VCE exhibited excellent diagnostic yield as the first-line diagnostic procedure. Push enteroscopy showed high value in both diagnosis of bleeding sites and performance of hemostatic interventions. In most cases, the bleeding can be identified on upper endoscopy and an intervention can be performed in more than one-half of the cases. We suggest additional studies comparing EGD and push enteroscopy in a head-to-head fashion as the initial procedure of choice in this cohort. LVAD as a destination therapy and use of any antiplatelet and warfarin were the top variables retained by the prediction algorithm in predicting risk of GI bleed in patients with LVAD. We propose a nomogram for use in clinical practice based on these variables to calculate the percent probability of GI bleed in this patient cohort. Further studies are warranted for the external validation of the proposed nomogram.

## Disclosure

All authors disclosed no financial relationships.

## Figures and Tables

**Supplementary Table 1:** Backward elimination of variables with evidence of multicollinearity using variance inflation factor (value of >10 indicates multicollinearity)

All introductory variables	Variables with positive association with GI bleed on binary logistics regression screen	First model	Second model (on any antiplatelet not included)	Third model (on any anticoagulant not included)
On any anticoagulant	Yes	18.06	10.99	x
On any antiplatelet	Yes	13.32	x	8.11
On warfarin	Yes	9.50	8.87	6.66
On DAPT	Yes	9.26	9.25	9.25
On dipyridamole	Yes	6.42	6.41	6.42
On clopidogrel	Yes	4.03	4.02	4.03
On heparin	Yes	2.67	2.54	2.12
On enoxaparin	Yes	1.10	1.09	1.04
Pulsatile or continuous flow	No	x	x	x
Age groups	Yes	1.02	1.02	1.02
Device types	No	x	x	x
Duration of LVAD device	No	x	x	x
Length of stay	No	x	x	x
Sex	Yes	1.03	1.03	1.02
Implant to discharge time	No	x	x	x
Implant indications	Yes	1.03	1.02	1.02

*DAPT*, Dual antiplatelets; *LVAD*, left ventricular assist device; *x,* not applicable.

**Supplementary Table 2:** Penalized regression coefficients of predictors retained in the LASSO model to predict GI bleeding after LVAD implantation

Predictors	Model derived using LASSO regression (N = 491)
Age groups	
18-34 y	–.397
34-49 y	…
50-64 y	…
65-79 y	…
≥80 y	…
Sex	
Female	.038
Male	…
LVAD implantation to discharge	
0-30 d	…
31-60 d	…
≥61 d	…
LVAD implant to discharge duration, median (IQR)	…
Duration of LVAD implantation, mo	…
Duration of LVAD implantation, median (IQR), d	…
LOS for LVAD admission, median (IQR), d	…
Continuous or pulsatile flow LVAD	
Continuous	…
Pulsatile	…
On any anticoagulant on admission	…
Type of anticoagulant on admission	
Warfarin	–1.94
Heparin	…
Enoxaparin	…
Indication for LVAD transplantation	
Bridge to decision	…
Bridge to recovery	…
Bridge to transplantation	–.280
Destination therapy	.41
Any antiplatelet use on admission for GI bleed	–4.22
DAPT on admission for GI bleed	…
Clopidogrel/ticagrelor/prasugrel use on admission for GI bleed	…
Dipyridamole use on admission for GI bleed	…
Median hemoglobin on admission (IQR), g/dL	…
Median INR on admission (IQR)	…
Total no. of predictors retained from the 10 in the model	6
Intercept	3.76
Mean Lambda	.02

*LASSO*, Least absolute shrinkage and selection operator; *LVAD*, left ventricular assist device; *IQR*, interquartile range; *LOS*, length of stay; *DAPT*, dual antiplatelets; *INR*, international normalized ratio.

**Supplementary Table 3:** Assessment of variables selected by LASSO model and further backward elimination of negative predictors

Variable	Regression coefficients	Standard error	z	*P*>|z|	95% Confidence interval
On any antiplatelets	4.60	.69	6.59	.000	3.238417 to 5.979351
On warfarin	2.38	.79	2.99	.003	.8231344 to 3.955319
Bridge to transplantation	–.46	.50	–.94	.348	–1.451807 to .512079
Destination therapy	.56	.50	1.26	.206	–.3089047 to 1.431455
Female sex	–.28	.47	.60	.552	–.6422028 to 1.202573
Age <34 y	–.95	.78	–1.21	.227	–2.495021 to .5921174
Constant	–2.49	.37	–6.60	.000	–3.230451 to –1.75079

Log likelihood = –119.01; no. of observations = 4.80; *probability* > χ^2^ = .0000; pseudo *R*^2^ = .5724.

*LASSO*, Least absolute shrinkage and selection operator.

**Supplementary Table 4:** Assessment of variables after backward elimination for nomogram development

Variables	Regression coefficients	Standard error	z	*P*>|z|	95% Confidence interval
On any antiplatelets	4.54	.685	6.64	.000	3.205069 to 5.890809
On warfarin	2.42	.80	3.00	.003	.8421173 to 4.014989
Destination therapy	.80	.36	2.24	.025	.1012232 to 1.516259
Constant	–2.80	.27	–10.14	.000	–3.348577 to –2.264021

Log likelihood = –118.88; no. of observations = 4.80; *probability* > χ^2^ = .0000; pseudo *R*^2^ = .5729.

## Appendix 1: Details of statistical analysis and supervised machine learning

Descriptive rates of GI bleeding outcomes of interest and endoscopic data were calculated as percentages. Statistical analysis was performed by using STATA version 16 (StataCorp LLC, College Station, Tex, USA). The derivation of a risk scoring system was divided into 4 steps. The first step used binary logistic regression to assess the direction of association of predictors with GI bleed in left ventricular assist device (LVAD) patients (Supplementary Table 1). The second step included evaluation of multicollinearity of shortlisted variables that showed a positive association with GI bleed in the previous step. Backward elimination was done for variables associated with multicollinearity, and the best model was selected for penalized regression. The third step included assessing the individual associations of shortlisted predictors and the exclusion of poor predictors using regression coefficients (Supplementary Table 2). The remaining predictors were used to develop a nomogram. The final step included k-fold cross-validation and assessing the model’s goodness of fit. A two-sample Wilcoxon rank sum (Mann-Whitney) test was used for continuous variables. The χ^2^ test was used to compare categorical variables.

### Supervised machine learning

We used the least absolute shrinkage and selection operator (LASSO) penalized regression for determining the adequate predictors (after multicollinearity assessment) of GI bleeding in LVAD patients. LASSO selects a subset of predictors by shrinking the coefficients of the minor contributive variables to zero, resulting in their exclusion. The tuning parameter lambda determines the coefficient shrinkage.^1^ Individual associations of the LASSO selected variables were evaluated, and poor predictive variables (regression coefficient in negative) were excluded.

### Nomogram development

Multivariate binary logistic regression was implemented for nomogram generation using the “nomolog” command in STATA for the finalized predictors. For the assessment of discriminative power, 10-fold cross-validation was used to generate receptor-operated curves. It prevents excessively optimistic estimates of predictive performance in overlooked cases; therefore, generating a pragmatic estimation of the final model’s predictive performance (area under the curve [AUC]) in new cases.^1^ It also delivers a lower bias of estimates than split-sample validation, resulting in more accurate predictions. The AUC value <.5 indicates no discrimination, .7 to .8 indicates acceptable, .8 to .9 excellent, and >.9 indicates outstanding predictive power.^2^

We also generated bias-corrected bootstrap 95% confidence intervals for the AUC. The agreement between the model-predicted and actual risk of GI bleeding in LVAD patients was evaluated via calibration belt plots. This allowed a visual inspection of any significant differences of observed frequencies compared with expected probabilities, as well as the direction of miscalibration.^3^ A calibration test indicating any significant deviations from the bisector (45 degree line of perfect fit) were also included in the calibration belt plot.^4^^,^^5^ The performance of the calibration belt is superior to that of the Hosmer-Lemeshow C test for assessing goodness of fit.^4^ The Brier score (lower is better), a measurement of variance between the predicted and the observed outcome, was used to assess overall performance.^2^
